# Drugs, Metabolites, and Lung Accumulating Small Lysosomotropic Molecules: Multiple Targeting Impedes SARS-CoV-2 Infection and Progress to COVID-19

**DOI:** 10.3390/ijms22041797

**Published:** 2021-02-11

**Authors:** Markus Blaess, Lars Kaiser, Oliver Sommerfeld, René Csuk, Hans-Peter Deigner

**Affiliations:** 1 Institute of Precision Medicine, Medical and Life Sciences Faculty, Furtwangen University, Jakob-Kienzle-Str. 17, D-78054 Villingen-Schwenningen, Germany; markus.blaess@web.de (M.B.); La.Kaiser@hs-furtwangen.de (L.K.); 2 Institute of Pharmaceutical Sciences, University of Freiburg, Albertstraße 25, D-79104 Freiburg, Germany; 3 Department of Anaesthesiology and Intensive Care Medicine, Jena University Hospital, Am Klinikum 1, D-07747 Jena, Germany; Oliver.Sommerfeld@med.uni-jena.de; 4 Organic Chemistry, Martin-Luther-University Halle-Wittenberg, Kurt-Mothes-Str. 2, D-06120 Halle (Saale), Germany; rene.csuk@chemie.uni-halle.de; 5 Fraunhofer Institute IZI, Leipzig, EXIM Department, Schillingallee 68, D-18057 Rostock, Germany; 6 Faculty of Science, Tuebingen University, Auf der Morgenstelle 8, D-72076 Tübingen, Germany

**Keywords:** SARS-CoV-2, COVID-19, lysosomotropism, metabolites, cytokine storm, viral host cell entry, approved drugs, pulmonary tissue accumulation, drug repurposing, eligibility criteria

## Abstract

Lysosomotropism is a biological characteristic of small molecules, independently present of their intrinsic pharmacological effects. Lysosomotropic compounds, in general, affect various targets, such as lipid second messengers originating from lysosomal enzymes promoting endothelial stress response in systemic inflammation; inflammatory messengers, such as IL-6; and cathepsin L-dependent viral entry into host cells. This heterogeneous group of drugs and active metabolites comprise various promising candidates with more favorable drug profiles than initially considered (hydroxy) chloroquine in prophylaxis and treatment of severe acute respiratory syndrome coronavirus 2 (SARS-CoV-2) infections/Coronavirus disease 2019 (COVID-19) and cytokine release syndrome (CRS) triggered by bacterial or viral infections. In this hypothesis, we discuss the possible relationships among lysosomotropism, enrichment in lysosomes of pulmonary tissue, SARS-CoV-2 infection, and transition to COVID-19. Moreover, we deduce further suitable approved drugs and active metabolites based with a more favorable drug profile on rational eligibility criteria, including readily available over-the-counter (OTC) drugs. Benefits to patients already receiving lysosomotropic drugs for other pre-existing conditions underline their vital clinical relevance in the current SARS-CoV2/COVID-19 pandemic.

## 1. Introduction

Severe acute respiratory syndrome coronavirus 2 (SARS-CoV-2) has been identified as the disease-causing pathogen of the pandemic Coronavirus disease 2019 (COVID-19) outbreak, posing serious challenges to health care systems worldwide. The most recent COVID-19 treatment guidelines published by the National Institutes of Health (NIH) [1] state that at present there is still no drug proven to be safe and effective for pre-exposure prophylaxis of SARS-CoV-2 infection. To date, dexamethasone and the recently approved nucleotide prodrug remdesivir are recommended for hospitalized COVID-19 patients requiring supplemental oxygen or noninvasive ventilation, and dexamethasone in case of extracorporeal membrane oxygenation (ECMO) or invasive mechanical ventilation. Moreover, the NIH meanwhile discourages the previously hotly-favored use of anti-IL-6 receptor antibodies (e.g., sarilumab, tocilizumab) or anti-IL-6 antibody (siltuximab), and ivermectin, except in a clinical trial [1]. There is still a need for action in the field of individual prophylaxis of SARS-CoV-2 infection and therapy of COVID-19 because of the rapidly increasing number of cases.

Lately, lysosomes have gained increasing attention in drug repurposing since they are considered to function as the key cell organelle involved in the SARS-CoV-2 infection of nasal and pulmonary tissue [2,3,4,5,6]. Repurposing of approved active compounds, with particular regard to their metabolites accumulating in the lysosomes of pulmonary tissue and mononuclear cells, could be a strategy to bridge this gap and to achieve a better outcome, unlike (hydroxy) chloroquine administration in COVID-19 [1] and SARS [7]. On the other hand, the use of these active compounds in the treatment of preexisting diseases, such as bacterial or fungal infections, mental illness, allergies, and hypertension, could be one reason for the variation in severities of the pandemic in distinct industrial countries.

## 2. (Endo) Lysosomes, Viral Replication, and Infection of Airway Epithelial Cells Are Therapeutic Targets of Small Lysosomotropic Molecules

Both SARS-CoV and SARS-CoV-2 engage their receptor, angiotensin-converting enzyme 2 (ACE2), on the cell surface for host cell entry. If human airway trypsin-like protease [8] is not expressed on host cell surface, both enter the cytoplasm through endosomes and travel along the endocytic pathway. Finally, in lysosomes active cathepsin L (BRENDA:EC 3.4.22.15; optimum pH 5.0–5.5) induces the fusion of SARS particles bound to ACE2 with host cells by cleavage of the viral S-protein [9]. Cathepsin L-induced fusion of SARS-CoV particles with host cells can be impeded via preventing (endo)lysosomal acidification by vacuolar H^+^-ATPase (V-ATPase; BRENDA:EC 3.6.3.6) using bafilomycin or via lysosomotropism and unspecific cathepsin L inhibition using lysosomotropic teicoplanin [10]. Moderate lysosomotropic compounds, such as chloroquine, hydroxychloroquine, fluspirilene, and clomipramine, have been found to abolish SARS-CoV-2 infection in Vero E6 cells [11,12,13]; chloroquine exerts antiviral SARS-CoV effects in vitro during pre- and post-infection conditions, impairs the terminal glycosylation of ACE2 at anti-SARS-CoV concentrations, and inhibits viral entry.

Interestingly, SARS-CoV-2 is capable of directly infecting cells by an infected neighboring cell without the release of a complete virus from infected cells via exocytosis. S-protein-related formation of multinucleate cells (syncytia) depends on presentation of ACE2 on the surface of the neighboring host cell. Syncytia has been observed in SARS-CoV-infected cell cultures, primates, and SARS patients [14] and is probably linked to lysosomal ceramide metabolism and lysosomal C_16_-/C_18_-ceramide synthesis. These findings suggest that both prophylactic and therapeutic effects lysosomotropic compounds in general are likely and are summarized in Table 1.

Transmembrane serine protease 2 (TMPRSS2), a member of the transmembrane protease/serine subfamily (TMPRSS), has been reported to induce SARS-CoV-2 fusion (S-protein priming) with membranes of airwave epithelial cells [8]. Cell culture experiments revealed, however, that TMPRSS2 inhibitors are only efficient in SARS-CoV-2 infection if the viral host cell entry is exclusively mediated by TMPRSS2. Once the cathepsin L-dependent endocytic, ACE2 receptor-mediated pathway of infection participates, the effectiveness of TMPRSS2 inhibitors (e.g., nafamostat) decreases rapidly [15] and becomes unpromising for drug repurposing.

**Table 1:** Potential scope of approved lysosomotropic drugs, RNA-dependent RNA polymerase (RdRp) severe acute respiratory syndrome coronavirus 2 (SARS-CoV-2) antivirals (remdesivir), and transmembrane serine protease 2 (TMPRSS2) inhibitors (nafamostat, camostat), in the prophylaxis and severity of SARS-CoV-2 infection/COVID-19. Depending on the impact on downstream signaling pathways/effects and on the disease process, the drug interventions are classified as indicated: ++/+ with putative (very) strong impact; o with putative low/moderate impact; − with putative no impact; * inhibits serine proteases related to coagulation and fibrinolysis and prevents (sepsis-related) disseminated intravascular coagulation and thrombotic microangiopathy; sufficient TMPRSS2 inhibiting concentration questionable in lung tissue within the therapeutic margin; # proven in animals (SARS-CoV-2) [16] or observation in psychiatry hospitals [17].

Prophylaxis/Severity of Disease	Impact of Lysosomotropic Compounds	SARS-CoV-2 RdRp Antivirals (Remdesivir)	TMPRSS2 Inhibitors
Pre-Exposure Prophylaxis	+/#	+/#	+/°
Asymptomatic or Pre-Symptomatic Infection	o	o	o
Mild Illness	+	+	−/*
Severe Illness	++	++	−/*
Critical Illness	++	++	−/*

The coloring underlines the severity of the disease / condition of the patient.

## 3. Tackling the CRS/Cytokine Storm Syndrome in COVID-19

SARS-CoV-2 is likely to cause pulmonary and systemic inflammation, thus leading to multi-organ dysfunction (e.g., acute respiratory distress syndrome (ARDS), myocarditis, and sepsis). The increase of systemic production and the substantial release of IL-8, TNFα, and IL-6 [18,19] have been considered central mediators of toxicity in cytokine release syndrome (CRS)/cytokine storm syndrome [20] and contribute to the pathophysiology of severe COVID-19 [21]. ARDS/severe COVID-19 pneumonia is often accompanied by the macrophage activation syndrome (MAS) or an IL-6-mediated very low human leukocyte antigen D related (HLA-DR) expression; a profound depletion (lymphopenia) of CD4 lymphocytes, CD19 lymphocytes, and natural killer (NK) cells; and hyper-inflammation [19].

In monocytic cells, the lysosmotropic model compound NB 06 diminishes LPS-induced surge of the prominent inflammatory messengers IL-1B; IL-23A; CCL4; CCL20; and, in particular, IL-6 [22]; likewise, beneficial effects in (systemic) infections involving bacterial endotoxins, such as LPS, by targeting the TLR4 receptor pathway in sepsis are obvious. Moreover, lysosomotropic desipramine reduces endothelial stress response in systemic inflammation owing to peritoneal infection [23]. Comparable high expression of IL-1B, IL-6, CCL4, CXCL10, and highly elevated serum levels of IL-6 have been observed in murine lung lobes infected with influenza A (H1N1) virus [24].

## 4. Lysosomotropic Drugs vs. IL-1 and IL-6 Inhibitors or Anti-IL-6R in COVID-19 Treatment

**Figure 1:**
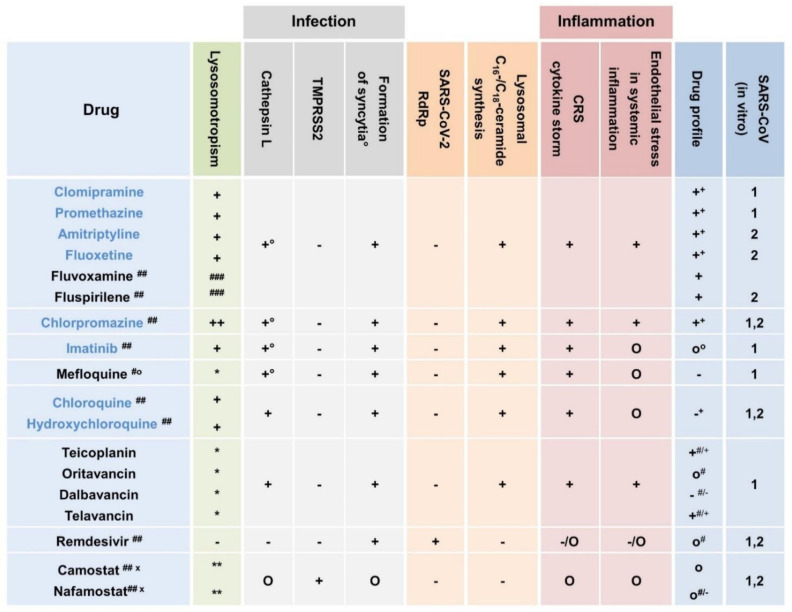
Variety of approved compounds for targets in SARS-CoV-2 infection/Coronavirus disease 2019 (COVID-19). Lysosomotropism: achievement of the desired lysosomotropic effect depended on the drug, the dosage, and accumulation in pulmonary tissue and in lysosomes. Unless indicated, maximum daily doses are typically split into three applications according to the patient information leaflet/market authorization. Confirmed lysosomotropism (blue colored): lysosomal drug concentration (effect) within the therapeutic margin in vivo (expected): (++) occurs at maximum daily dosage and very likely in low or initial dosage, (+) very likely at maximum daily dosage and possible in low or initial dosage. Supposed lysosomotropism: (*) lysosomotropism very likely but not yet confirmed, lysosomal drug concentration (effect) within the therapeutic margin expected; (**) lysosomotropism likely but not yet confirmed, lysosomal drug concentration (effect) within the therapeutic margin not expected; (^###^) lysosomotropism very likely but not yet confirmed, no lysosomal drug concentration (effect) within the therapeutic margin expected. Efficacy on targets cathepsin L, TMPRSS2, formation of syncytia, SARS-CoV-2 RdRp, lysosomal C_16_-/C_18_-ceramide synthesis, endothelial stress in systemic inflammation, and CRS/cytokine storm [8,10,11,12,22,25,26,27,28,29]: (+°) proven in vitro; (+) is very likely; (O) is (mediated) possible; (-) no effect. Drug profile: (+) good; (o) moderate; (-) worse, assessment depending on elimination half-life and severe adverse effects; accumulation in pulmonary tissue: (^+^) proven, (^o^) likely, (**^-^**) no accumulation in pulmonary tissue; (^#^) only intravenous application; (^##^) in COVID-19 clinical trials; (^#o^) dosage depending on treatment or prophylaxis (of malaria); * inhibits serine proteases related to coagulation and fibrinolysis, and prevents (sepsis-related) disseminated intravascular coagulation and thrombotic microangiopathy; (^x^) sufficient TMPRSS2 inhibiting concentration questionable in pulmonary tissue within the therapeutic margin.

Various clinical trials on the use of immunomodulatory IL-1 and IL-6 inhibitors or anti-IL-6R antibodies (anakinra, tocilizumab, and sarilumab) in patients with COVID-19 are currently under way (https://clinicaltrials.gov/ct2/home). In limited yet available clinical data, tocilizumab, as well as sarilumab, have failed to demonstrate efficacy and benefit in survival. The NIH, therefore, discourages the use of IL-6 or IL-6 receptor antibodies, except in a clinical trial [1], considering the risk of serious adverse effects due to massive interventions in the immune system (e.g., bacterial upper respiratory tract infection, pneumonia, and viral infections). As a way out of this dilemma, it would be preferable to prevent the excessive emergence of this messenger by clever selection of lysosomotropic drugs rather than to trap them after excessive release. Some benefits and targets that are not addressed with antibodies but are with lysosomotropic drugs are enumerated below (Figure 1).

First of all, lysosomotropic compounds are supposed to tackle the CRS/cytokine storm syndrome in transition to COVID-19. Second, they prevent the required lysosomal acidification for fusion-activating cathepsin L activity to diminish or prevent airwave cell infection by releasing viral RNA. Third, lysosomotropic compounds have an impact on lysosomal ceramide metabolism. Of particular interest is an assumed exocytosis-triggering effect of C_18_-ceramide [30], leading to cell–cell fusion of fractions of viral S-protein and formation of syncytia. Lysosomotropic drugs prevent apoptosis and lysosomal synthesis of C_16_-ceramide [22], and are supposed to prevent exocytosis-triggering C_18_-ceramide. Fourth, lysosmotropic drugs provide protection against endothelial stress response in systemic inflammation and sepsis-induced cardiac dysfunction [23]. Fifth, active compounds of various indications share lysosomotropic characteristics and partly display more suitable drug profiles than (hydroxy) chloroquine. Furthermore, their N-desmethyl metabolites share most likely lysosomotropism as well.

## 5. Lysosomotropism, Lysosomes, Lysosomal pH, NADPH, V-ATPase, and the Release of Virions

To date, an unanswered question is how the exocytosis of virions mechanistically occurs from infected cells. Non-lytic release of beta-coronaviruses, such as SARS-CoV-2, results in lysosome deacidification, inactivation of lysosomal degradation enzymes, and disruption of antigen presentation [31]. Conversely, it can be hypothesized that the lysosomal deacidification is necessary for the exocytosis of virions. Accordingly, C_18_-ceramide (and C_16_-ceramide) can be formed in lysosomes, triggering the formation of vesicles and exocytosis of virions.

The key point is the interaction of lysosomal acidic ceramidase (aCERase), the lysosomal vacuolar H^+^-ATPase (V-ATPase), and NADPH. Interestingly, lysosomal acidic ceramidase (aCERase) exhibits two different intergradient enzymatic activities depending on the lysosomal pH: a ceramide hydrolase activity (optimum pH 4.0–5.0) and a little-known ATP-independent reverse ceramide synthase activity (revaCERase) (optimum pH 5.5–6.5). RevaCERase preferentially reacts palmitic acid (C_16_) and stearic acid (C_18_) with sphingosine [32,33]. In the event of oxidative stress, stearic acid and palmitic acid are derived from palmitoyl-CoA or stearoyl-CoA by palmitoyl-CoA hydrolase from fatty acid synthesis, if ELOVL-catalyzed elongation to very long chain fatty acids is impeded due to NADPH deficiency. NADPH serves as an activator of ELOVL 6 and 7 [34].

Deficiency of cytosolic NADH and ATP, e.g., in oxidative stress, collapses the lysosomal proton gradient powered by the lysosomal redox chain and the V-ATPase [35,36]. Under such conditions, the short-chain ceramides C_16_-ceramide and C_18_-ceramide can be formed.

However, in absence of cellular oxidative stress, neither C_16_-ceramide nor C_18_-ceramide are formed in lysosomes [37]; elongation of palmitoyl-CoA and stearoyl-CoA to very long chain fatty acids is not impeded. In presence of lysosomotropic compounds, the ongoing synthesis of long-chain ceramides leads to an accumulation of long-chain ceramides (e.g., C_24:1_-ceramide) [22] without triggering vesicle formation [30].

## 6. Chlorpromazine: Pulmonary Tissue Accumulation and Protection from SARS-CoV-2 Infection/COVID-19

**Figure 2:**
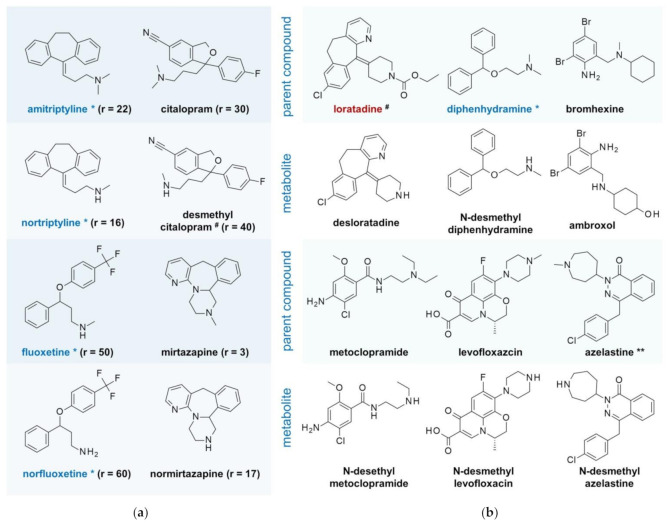
Lysosomotropic candidates and pulmonary tissue accumulation: (**a**) pairs of confirmed (blue colored) and potential lysosomotropic drugs and their N-desmethyl metabolites with proved enrichment in pulmonary tissue (ratio of pulmonary tissue/plasma concentration r > 1) [40,41,42]. (^#^) Ratio determined in rats, (*) lysosomotropism is confirmed [26]; (**b**) candidates for (systemic) prophylaxis of viral infection and transition to COVID-19. The over-the-counter drugs bromhexine, ambroxol, loratadine, desloratadine, azelastine, and diphenhydramine are assumed to contribute significantly to viral infection prophylaxis and frequency in less affected countries such as Germany. Unless indicated, lysosomotropism is highly probable, but not confirmed. (*) lysosomotropism is confirmed, (^#^) no lysosomotropism, (**) nasal and ophthalmic H_1_-antihistamine. Levofloxacin exhibits anti-inflammatory effects on prominent inflammatory messengers in mice with LPS-induced acute lung injuries [43], comparable to NB 06 in LPS-induced inflammation [22].

Predicted protective effects of particular lysosmotropic drugs in SARS-CoV-2 infection/COVID-19 are supported by observations in patients treated with chlorpromazine. Patients displayed a significantly lower prevalence of symptomatic and severe forms of COVID-19 (4%) compared to staff (14%) operating in the same facilities [17]. Single dose distribution experiments demonstrated a lasting enrichment of chlorpromazine in pulmonary tissue (tissue/plasma ratio of 30) 60 min after intravenous application [38]. In line with in vitro data of chlorpromazine [12,27], various lysosomotropic drugs (Figure 2a,b) are likely to provide anti SARS-CoV(-2) effects in patients if pulmonary enrichment leads to an effective antiviral (lysosomotropic drug) concentration in vivo.

Despite the aforementioned protection, chlorpromazine is inappropriate for systemic prophylaxis of infection in healthy individuals. The intrinsic pharmacological effects of chlorpromazine now turn into undesirable adverse effects (e.g., dry mouth, bloating or stomach cramps, and feeling restless [39]) in people without mental illness. A reasonable solution of this quandary is the inhalation of chlorpromazine-containing-solutions for SARS-CoV-2 infection prophylaxis, as this enables us to accurately target the area of viral host cell entry.

## 7. Fluvoxamine: Tackling Dyspnea in COVID-19

Fluvoxamine is another approved drug with a molecular structure suggesting lysosomotropism that has been recently successfully tested for its efficacy in COVID 19. One hundred and fifty-two adult outpatients with confirmed COVID-19 and symptom onset within day seven were treated with fluvoxamine (up to 300 mg/day) and compared with placebo. The randomized preliminary clinical trial demonstrated a statistically significant lower likelihood of clinical deterioration (low oxygen saturation (<92%) and presence of dyspnea, or hypoxia) over 15 days (0 vs. 6 (8.3%) with placebo (*n* = 66)) [44]. In general, fluvoxamine is a well-tolerated, selective serotonin reuptake inhibitor (SSRI) with a broad therapeutic window (lower QT-prolonging potential [45]) used daily in doses up to 300 mg. Moreover, fluvoxamine is rated as a strong σ-1 receptor (S1R) agonist, reducing the damaging aspects of the inflammatory response during sepsis through the S1R-IRE1 pathway and decreasing shock in murine sepsis models [46] similar to lysosomotropic desipramine [23]. This, together with its strong lipophilicity and rapid intracellular uptake [47], supports distinct lysosomotropic characteristics of fluvoxamine.

Although the adverse events during the clinical trial have been moderate, the incidence of serious drug interactions, characteristic psychiatric adverse reactions of SSRI (e.g., trouble sleeping, insomnia (up to 35%), mood or behavior changes, anxiety, and panic attacks), and dermatologic adverse reactions (skin rash, blisters, or hives) [48] can be reasonably expected to increase with large-scale and high-dose usage.

## 8. Drug Repurposing Lessons Learned from (Hydroxy) Chloroquine in Clinical Trials

Both chloroquine and hydroxychloroquine were demonstrated in cell culture experiments to prevent SARS-CoV-2 infection [11,49,50]; however, they have failed to demonstrate their benefits in clinical trials, leading to a dissuasion from the application [1]. Compounds and their metabolites (e.g., chloroquine and desethylchloroquine) with (very) long terminal elimination half-life (45 ± 15 days chloroquine [51,52,53], 41 ± 11 days hydroxychloroquine [53,54]), and poor and/or delayed pulmonary accumulation (steady state on day 10 [55]) of both compounds are inappropriate in terms of adverse effects (e.g., dysrhythmias, often occurring in combination with other drugs such as azithromycin (lysosomotropism presumed), prolonging the QTc interval in and beyond the therapeutic window [1,56,57]).

In particular, the high dosage of, e.g., 600 mg hydroxychloroquine daily for 7 days in the treatment of (mild) COVID-19 [58], in combination with the long elimination half-life of both compounds, 30–60 days and varying substantially from one person to another, impedes therapy management and increases the risk of unacceptable serious adverse effects. Oxidative stress resulting from SARS infection in animal models of [59] implies a high risk of serious adverse reactions such as hemolysis and methemoglobinemia in glucose-6-phosphate dehydrogenase (G6PD)-deficient patients, if treated with (hydroxy) chloroquine [60].

The disappointing results of (hydroxy) chloroquine contrast with the encouraging clinical data of chlorpromazine [17] and fluvoxamine [44]. All of them have lysosomotropism in common; however, they differ in their drug profile. This encourages a quest for alternative lysosomotropic drugs with a more suitable drug profile (lower elimination half-life and lower dosage—required for lysosomotropic drug concentration in pulmonary tissue—better G6PD tolerance, a broader therapeutic window, and lower (cardiac) toxicity).

## 9. Quest for Further Lysosomotropic (Active) Compounds

Lysosomotropism is a noteworthy biological characteristic of small molecules, independently present in addition to their intrinsic pharmacological effects. Various well-known approved drugs, e.g., amitriptyline, amlodipine, chlorpromazine, doxepine, and sertraline [26], share lysosomotropic characteristics. In silicio, search methods such as the SPAR model [26] or the QSAR model [5] are useful tools to identify existing highly accumulating lysosomotropic drugs. Their shortcoming, however, is that the search is focused on well-known drugs, neglecting resulting active metabolites and the respective bioavailability as well as accumulation in pulmonary tissue. The pair consisting of the parent compound loratadine (no lysosomotropism) [26] and the metabolite desloratadine (lysosomotropism, anti-inflammatory in mice lungs) [61] is an example of the shortcoming of both models.

## 10. Metabolites and Lung Accumulation

Investigations by toxicologists on the accumulation of various psychotropic drugs in lung tissue have provided interesting results regarding lysosomotropic drugs and their major metabolites. Both parent compounds and N-desmethyl metabolites of, e.g., fluoxetine, citalopram, mirtazapine, and amitriptyline, accumulate in pulmonary tissue. Most commonly, the metabolites and the parent compounds are hardly structurally different (e.g., fluoxetine/norfluoxetine, citalopram/desmethyl citalopram) (Figure 2a). Normirtazapine, on the contrary, accumulates more than five times more in the lung tissue than mirtazapine. By taking advantage of lysosomal trapping and enrichment, a lysosomotropic drug concentration in pulmonary tissue and thus a preventive effect analogous to chlorpromazine can be achieved with, e.g., multiple low therapeutic dosing of mirtazapine.

## 11. Targeted Drug Repurposing

**Figure 3:**
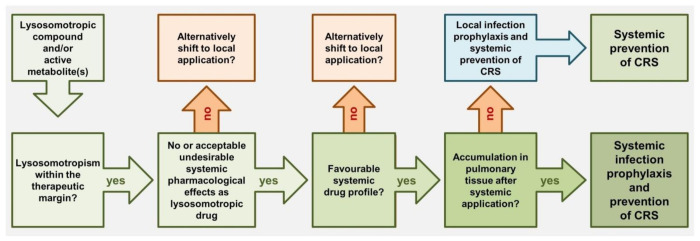
Flowchart for choosing suitable lysosomotropic drugs for (systemic) infection prophylaxis and prevention of transition to COVID-19 according to Table 1. Candidates (active compounds and/or one or more metabolites) should meet the following criteria: lipophilic, weak protonatable nitrogen bases where lysosomotropism is present within the therapeutic margin, no or acceptable undesirable systemic pharmacological effects as lysosomotropic drug are known, favorable systemic drug profile or local application as expedient, and accumulation in pulmonary tissue after systemic application.

Starting from the aforementioned findings, a new strategy for rational drug targeting can be derived for candidate drugs affecting the endolysosomal pathway of viral infection and providing anti-inflammatory effects in pulmonary tissue and, thus, in vivo. The flowchart in Figure 3 may be helpful to choose suitable lysosmotropic drugs and/or precursors of lysosomotropic metabolites meeting these requirements. Candidates are lipophilic-weak at pH 4.5–5.5 proton able bases containing at least one aliphatic (cyclic) amine residue meeting the following criteria (according to [22,26,62]): lysosomotropism is present within the therapeutic margin, no or acceptable undesirable systemic pharmacological effects at lysosomotropic drug concentrations, favorable systemic drug profile or local application as expedient, and accumulation in pulmonary tissue after systemic application. The lack of lysosomotropism of 2-hydroxy and 10-hydroxy derivatives of imipramine [26] suggests that hydroxyl group containing metabolites are unlikely to provide benefit in this context.

## 12. Candidates for (Systemic) Prophylaxis of Viral Infection and Transition to COVID-19

The pairs in Figure 2a,b are, in general, new promising drugs (and precursors) for prophylaxis and treatment of SARS-CoV-2 infection of airway epithelial cells. Like chlorpromazine, the psychotropic drugs mirtazapine, normirtazapine, amitriptyline, nortriptyline, fluoxetine, and citalopram, and the antiemetic metoclopramide, are inappropriate for systemic prophylaxis in healthy individuals.

In contrast, the OTC drugs bromhexine/ambroxol, diphenhydramine, and loratadine, by its active metabolite desloratadine, may provide a substantial contribution to prophylaxis and systemic therapy and the approved nasal applicable azelastine to nasal prophylaxis. Their therapeutic window is broader, the drug profile is safer, and the generic adverse effects of lysosomotropic prescription drugs (e.g., QTc prolongation, Torsade de Pointes, ventricular arrhythmia, bundle branch heart block, and cardiac deaths, in particular from overdosing) are very rare (loratadine/desloratadine) [63] or absent (ambroxol) [64].

## 13. Defined Daily Doses (DDD) of Commonly Used (Presumable) Lysosmotropic Drugs and Prodrugs

However, since some of the drugs in Figure 2a,b are already and frequently used therapeutically, it is reasonable to assume that they may contribute significantly to viral infection prophylaxis in less affected countries such as Germany. In particular, ambroxol, loratadine, and its active metabolite desloratadine are widely used antiallergics in Germany. To gain an overview of the frequency of use of some of the listed candidates and the associated probable effects on the frequency of infection, the number of DDD prescriptions is a starting point. According to prescriptions charged to the statutory health insurance in Germany, amitriptyline (75 mg DDD/83.1 mio DDD), citalopram (20 mg/294.7), escitalopram (10 mg/110.5), fluoxetine (20 mg/62.2), mirtazapine (150 mg/185.4), sertraline (50 mg/143.0), and ciprofloxacin (1000 mg/19,3) (2016); promethazine (75 mg/31.4) (2017); and metoprolol (150 mg/834.9) (2018) were prescribed [65]. The data suggest that psychotropic drugs previously identified as lysosomotropic are widely used. Due to their piperazine residue, the antibiotics ciprofloxacin and levofloxacin are very likely to share lysosomotropic characteristics. They may be useful in bacterial pneumonia and show anti-inflammatory effects in LPS-induced acute lung injuries [43], such as NB 06 in LPS-treated monocytes [22], suggesting a common mode of action.

## 14. Conclusions

Encouraging results in vitro and of chlorpromazine and fluvoxamine in vivo suggest that lysosomotropic compounds might be used as tools in fighting SARS-CoV-2 infections by potentially triggering a variety of cellular modifications that impede alveolar cell infection and viral replication (Figure 1). The modulating effects of lysosomotropism of drugs and their metabolites on gene expression of various cytokines and interleukins is likely to provide a means of preventing the development of CRS and, concomitantly, the rapid, severe, and serious deterioration in COVID-19 without massive interventions in the immune system.

Lysosomotropism is present in an enormous number of active compounds and metabolites, but such activity has been confirmed only in a few so far. The eligibility criteria, lysosomotropism, drug profile, and inevitable lysosomal accumulation in pulmonary tissue described here (Figure 3) provide a guideline for picking promising candidates from the pool of approved drugs for future investigations and clinical trials. If further confirmed, the concept may contribute more rapidly accessible options for preventing SARS-Cov-2 infections, the transition to COVID-19, and providing protection of endothelial stress response in systemic inflammation. Moreover, further infectious diseases such as influenza A (H1N1) can be addressed, where pH in lysosomes, IL-6, and lysosomal sphingolipid metabolism play a crucial role.

## Data Availability

Not applicable.

## Abbreviations

ACE2	Angiotensin-converting enzyme 2
aCERase	lysosomal acidic ceramidase
ARDS	acute respiratory distress syndrome
COVID-19	Coronavirus disease 2019
CRS	cytokine release syndrome
DDD	defined daily dose
ELOVL	elongation of very long chain fatty acids protein
G6PD	glucose-6-phosphate dehydrogenase
HLA-DR	human leukocyte antigen-D related
IL-1R	IL-1 receptor
IL-6R	IL-6 receptor
IRE1	inositol-requiring enzyme 1α
LPS	lipopolysaccharide
RdRp	RNA-dependent RNA polymerase
SARS	severe acute respiratory syndrome
SSRI	selective serotonin reuptake inhibitor
TLR4	toll-like receptor 4
TMPRSS2	transmembrane serine protease 2, transmembrane protease serine 2
V-ATPase	vacuolar H^+^-ATPase
